# 5-Acetyl-3-(5-phenyl-1*H*-pyrazol-3-yl)-1,3,4-thia­diazol-2(3*H*)-one monohydrate

**DOI:** 10.1107/S1600536813010817

**Published:** 2013-04-27

**Authors:** Abdullah M. Asiri, Muhammad Nadeem Arshad, Abdullah Y. Obaid, Ghulam Mustafa

**Affiliations:** a Chemistry Department, Faculty of Science, King Abdulaziz University, PO Box 80203, Jeddah 21589, Saudi Arabia; b Center of Excellence for Advanced Materials Research (CEAMR), Faculty of Science, King Abdulaziz University, PO Box 80203, Jeddah 21589, Saudi Arabia; c Department of Chemistry, University of Gujrat, Gujrat 50700, Pakistan

## Abstract

In the title hydrate, C_13_H_10_N_4_O_2_S·H_2_O, the dihedral angles between the central pyrazole ring and its pendant phenyl and thia­diazole rings are 9.93 (8) and 4.56 (7)°, respectively. In the crystal, the components are linked by N—H⋯O, O—H⋯N and O—H⋯O hydrogen bonds, generating [100] chains incorporating *R*_4_^4^(10) loops. A weak C—H⋯O inter­action helps to consolidate the packing.

## Related literature

For the synthesis of the title compound, see: Abdelhamid *et al.* (2001 ▶). For a related structure, see: Ge (2006 ▶).
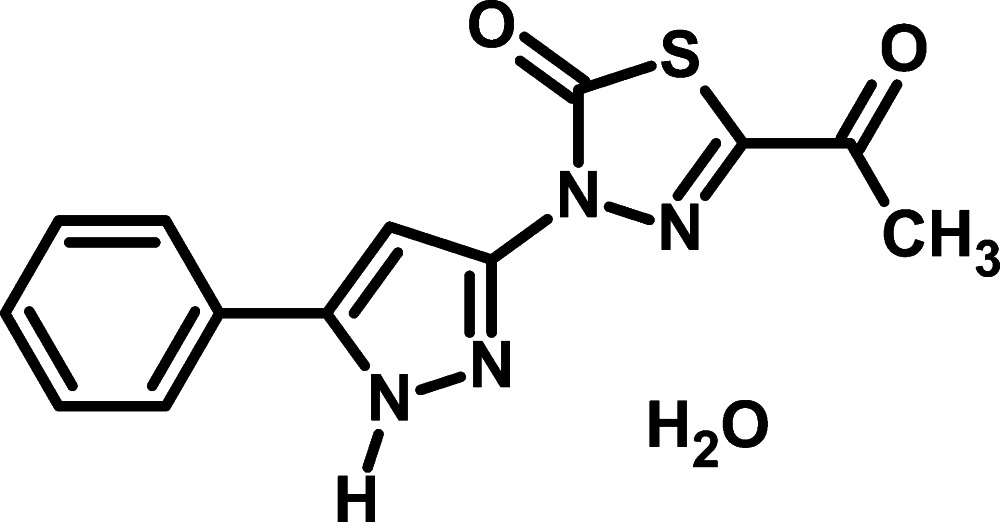


## Experimental

### 

#### Crystal data

C_13_H_10_N_4_O_2_S·H_2_O
*M* *_r_* = 304.33
Monoclinic, 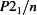

*a* = 7.6084 (2) Å
*b* = 25.5788 (4) Å
*c* = 7.6524 (2) Å
β = 111.974 (3)°
*V* = 1381.07 (6) Å^3^
*Z* = 4
Cu *K*α radiation
μ = 2.25 mm^−1^
*T* = 296 K
0.35 × 0.19 × 0.06 mm

#### Data collection

Agilent SuperNova (Dual, Cu at zero, Atlas, CCD) diffractometer
Absorption correction: multi-scan (*CrysAlis PRO*; Agilent, 2012 ▶) *T*_min_ = 0.778, *T*_max_ = 1.000
10797 measured reflections
2806 independent reflections
2498 reflections with *I* > 2σ(*I*)
*R*_int_ = 0.032

#### Refinement

*R*[*F* ^2^ > 2σ(*F* ^2^)] = 0.036
*wR*(*F* ^2^) = 0.106
*S* = 1.07
2806 reflections
238 parameters
All H-atom parameters refined
Δρ_max_ = 0.23 e Å^−3^
Δρ_min_ = −0.19 e Å^−3^

### 

Data collection: *CrysAlis PRO* (Agilent, 2012 ▶); cell refinement: *CrysAlis PRO*; data reduction: *CrysAlis PRO*; program(s) used to solve structure: *SHELXS97* (Sheldrick, 2008 ▶); program(s) used to refine structure: *SHELXL97* (Sheldrick, 2008 ▶); molecular graphics: *PLATON* (Spek, 2009 ▶); software used to prepare material for publication: *WinGX* (Farrugia, 2012 ▶) and *X-SEED* (Barbour, 2001 ▶).

## Supplementary Material

Crystal structure: contains datablock(s) I, global. DOI: 10.1107/S1600536813010817/hb7073sup1.cif

Structure factors: contains datablock(s) I. DOI: 10.1107/S1600536813010817/hb7073Isup2.hkl

Supplementary material file. DOI: 10.1107/S1600536813010817/hb7073Isup3.cml

Additional supplementary materials:  crystallographic information; 3D view; checkCIF report

## Figures and Tables

**Table 1:** Hydrogen-bond geometry (Å, °)

*D*—H⋯*A*	*D*—H	H⋯*A*	*D*⋯*A*	*D*—H⋯*A*
N1—H1⋯O3*W* ^i^	0.93 (2)	1.83 (2)	2.749 (2)	173 (2)
O3*W*—H3*A*⋯N2^ii^	0.86 (3)	1.99 (3)	2.8402 (19)	169 (2)
O3*W*—H3*B*⋯O1^iii^	0.83 (5)	2.19 (5)	2.995 (2)	163 (4)
C8—H8⋯O1^iv^	0.93 (2)	2.50 (2)	3.4100 (19)	167.8 (15)

Symmetry codes: (i) 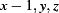; (ii) 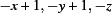; (iii) 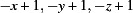; (iv) 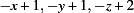.

**Fig. 1.**
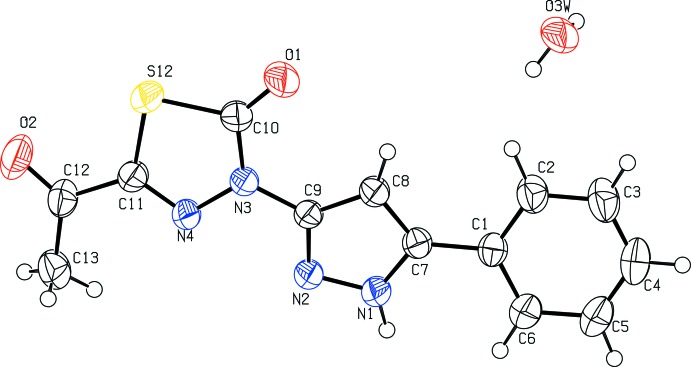
The molecular structure of title compound with 50% probability displacement ellipsoids.

**Fig. 2.**
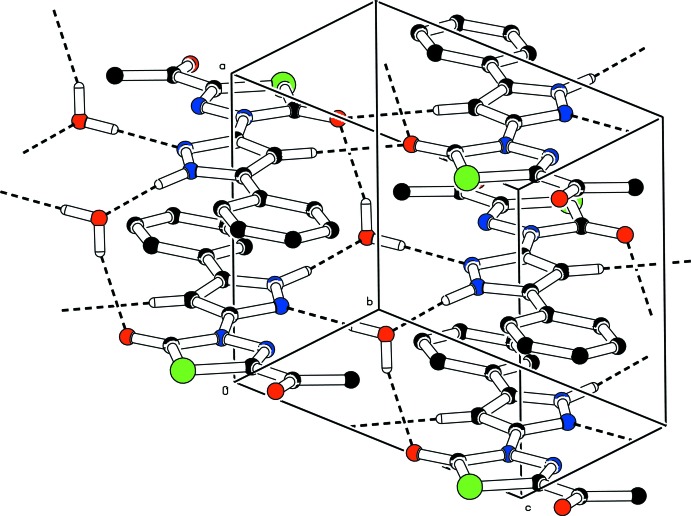
The packing diagram showing inversion dimers through hydrogen bonds, drawn using dashed lines.

## supplementary crystallographic information

### Comment

In the title compound (I), shown in Fig. 1, the aromatic ring is inclined at angles of 9.93 (8)° & 6.36 (7)° with respect to pyrazole and thiadiazole rings. The pyrazole and thiadiazole rings are oriented at dihedral angle of 4.56 (7)°. The acetyl group is inclined at dihedral angle of 6.00 (2)° with thiadiazole ring. The water molecule as usual is involved in classical hydrogen bonding interactions. The interactions through N1—H1···O3w, and O3w—H3a···N2 give rise to inversion dimers forming ten membered ring motif *R*_4_^4^(10). These dimers further connected through O3w—H3b···O1 interaction to make infinite one dimensional chain along *a* axis. Another non-classical interaction (C8—H8···O1) generates twelve membered ring motif *R*_1_^1^(12) loops; for symmetry detail, see; Table. 1, Fig. 2.

### Experimental

The title compound was synthesised according to literature (Abdelhamid *et al.*, 2001 ) procedure and recrystalized from methanol solution under slow evaporation to yield brown plates.

### Refinement

All the hydrogen atoms found from a Fourier difference map and allowed to refine freely with appropriate riding models.

### Crystal data

**Table:**

C_13_H_10_N_4_O_2_S·H_2_O	*F*(000) = 632
*M**_r_* = 304.33	*D*_x_ = 1.464 Mg m^−^^3^
Monoclinic, *P*2_1_/*n*	Cu *K*α radiation, λ = 1.54184 Å
Hall symbol: -P 2yn	Cell parameters from 5915 reflections
*a* = 7.6084 (2) Å	θ = 3.5–74.7°
*b* = 25.5788 (4) Å	µ = 2.25 mm^−^^1^
*c* = 7.6524 (2) Å	*T* = 296 K
β = 111.974 (3)°	Plate, brown
*V* = 1381.07 (6) Å^3^	0.35 × 0.19 × 0.06 mm
*Z* = 4	

### Data collection

**Table:**

Agilent SuperNova (Dual, Cu at zero, Atlas, CCD) diffractometer	2806 independent reflections
Radiation source: SuperNova (Cu) X-ray Source	2498 reflections with *I* > 2σ(*I*)
Mirror monochromator	*R*_int_ = 0.032
ω scans	θ_max_ = 74.8°, θ_min_ = 3.5°
Absorption correction: multi-scan (*CrysAlis PRO*; Agilent, 2012)	*h* = −9→9
*T*_min_ = 0.778, *T*_max_ = 1.000	*k* = −31→31
10797 measured reflections	*l* = −9→6

### Refinement

**Table:**

Refinement on *F*^2^	Primary atom site location: structure-invariant direct methods
Least-squares matrix: full	Secondary atom site location: difference Fourier map
*R*[*F*^2^ > 2σ(*F*^2^)] = 0.036	Hydrogen site location: inferred from neighbouring sites
*wR*(*F*^2^) = 0.106	All H-atom parameters refined
*S* = 1.07	*w* = 1/[σ^2^(*F*_o_^2^) + (0.0609*P*)^2^ + 0.1856*P*] where *P* = (*F*_o_^2^ + 2*F*_c_^2^)/3
2806 reflections	(Δ/σ)_max_ = 0.002
238 parameters	Δρ_max_ = 0.23 e Å^−^^3^
0 restraints	Δρ_min_ = −0.19 e Å^−^^3^

### Special details

**Table:**

Geometry. All esds (except the esd in the dihedral angle between two l.s. planes) are estimated using the full covariance matrix. The cell esds are taken into account individually in the estimation of esds in distances, angles and torsion angles; correlations between esds in cell parameters are only used when they are defined by crystal symmetry. An approximate (isotropic) treatment of cell esds is used for estimating esds involving l.s. planes.
Refinement. Refinement of F^2^ against ALL reflections. The weighted R-factor wR and goodness of fit S are based on F^2^, conventional R-factors R are based on F, with F set to zero for negative F^2^. The threshold expression of F^2^ > 2sigma(F^2^) is used only for calculating R-factors(gt) etc. and is not relevant to the choice of reflections for refinement. R-factors based on F^2^ are statistically about twice as large as those based on F, and R- factors based on ALL data will be even larger.

### Fractional atomic coordinates and isotropic or equivalent isotropic displacement parameters (Å^2^)

**Table:**

	*x*	*y*	*z*	*U*_iso_*/*U*_eq_	
S12	0.59223 (6)	0.644569 (15)	0.75319 (6)	0.05072 (15)	
O1	0.5175 (2)	0.55662 (5)	0.90067 (17)	0.0659 (4)	
N3	0.40123 (17)	0.56623 (4)	0.57705 (17)	0.0362 (3)	
O2	0.5725 (2)	0.72711 (5)	0.4716 (2)	0.0757 (4)	
N4	0.39508 (17)	0.60041 (4)	0.43842 (17)	0.0375 (3)	
N1	0.11453 (18)	0.46444 (5)	0.33689 (18)	0.0408 (3)	
N2	0.19673 (18)	0.51164 (5)	0.34177 (17)	0.0398 (3)	
C10	0.4991 (2)	0.58140 (6)	0.7612 (2)	0.0439 (3)	
C7	0.15808 (19)	0.44351 (5)	0.5095 (2)	0.0361 (3)	
C9	0.29698 (18)	0.51911 (5)	0.52301 (19)	0.0338 (3)	
C11	0.4876 (2)	0.64236 (5)	0.5106 (2)	0.0398 (3)	
C1	0.08453 (19)	0.39258 (5)	0.5408 (2)	0.0383 (3)	
C8	0.2800 (2)	0.47822 (5)	0.6365 (2)	0.0387 (3)	
C12	0.4979 (2)	0.68747 (6)	0.3918 (3)	0.0487 (4)	
C2	0.1125 (2)	0.37673 (7)	0.7226 (3)	0.0505 (4)	
H2	0.177 (3)	0.3992 (9)	0.834 (3)	0.068 (6)*	
C6	−0.0130 (2)	0.35941 (6)	0.3906 (3)	0.0480 (4)	
H6	−0.037 (3)	0.3691 (8)	0.264 (3)	0.052 (5)*	
C13	0.4163 (3)	0.68076 (8)	0.1839 (3)	0.0589 (5)	
H13B	0.364 (5)	0.7089 (14)	0.126 (5)	0.116 (11)*	
H13C	0.501 (6)	0.6663 (17)	0.145 (7)	0.165 (17)*	
H13A	0.313 (4)	0.6562 (12)	0.135 (4)	0.099 (9)*	
C4	−0.0469 (3)	0.29574 (6)	0.6054 (3)	0.0599 (5)	
H4	−0.085 (3)	0.2633 (9)	0.629 (3)	0.071 (6)*	
C3	0.0476 (3)	0.32821 (7)	0.7542 (3)	0.0600 (5)	
H3	0.071 (3)	0.3194 (9)	0.876 (4)	0.072 (7)*	
C5	−0.0788 (3)	0.31131 (6)	0.4244 (3)	0.0570 (4)	
H5	−0.140 (4)	0.2917 (9)	0.319 (4)	0.078 (7)*	
H1	0.025 (3)	0.4545 (8)	0.221 (3)	0.059 (6)*	
H8	0.337 (3)	0.4743 (7)	0.766 (3)	0.046 (5)*	
O3W	0.8281 (2)	0.44007 (6)	0.0009 (2)	0.0683 (4)	
H3A	0.816 (4)	0.4505 (10)	−0.110 (4)	0.089 (8)*	
H3B	0.722 (6)	0.4442 (16)	0.006 (5)	0.145 (16)*	

### Atomic displacement parameters (Å^2^)

**Table:**

	*U*^11^	*U*^22^	*U*^33^	*U*^12^	*U*^13^	*U*^23^
S12	0.0611 (3)	0.0453 (2)	0.0392 (2)	−0.01426 (16)	0.01118 (19)	−0.00717 (15)
O1	0.0838 (9)	0.0722 (8)	0.0307 (6)	−0.0271 (7)	0.0087 (6)	0.0042 (6)
N3	0.0429 (6)	0.0336 (5)	0.0290 (6)	−0.0032 (4)	0.0100 (5)	0.0016 (4)
O2	0.0992 (11)	0.0428 (6)	0.0740 (10)	−0.0249 (7)	0.0196 (8)	−0.0011 (6)
N4	0.0448 (6)	0.0321 (5)	0.0341 (6)	−0.0010 (4)	0.0131 (5)	0.0029 (4)
N1	0.0482 (6)	0.0376 (6)	0.0334 (7)	−0.0080 (5)	0.0116 (5)	−0.0008 (5)
N2	0.0483 (6)	0.0368 (6)	0.0322 (6)	−0.0070 (5)	0.0125 (5)	0.0003 (5)
C10	0.0480 (8)	0.0467 (8)	0.0335 (8)	−0.0085 (6)	0.0114 (6)	−0.0010 (6)
C7	0.0384 (6)	0.0333 (6)	0.0354 (7)	0.0021 (5)	0.0124 (6)	0.0029 (5)
C9	0.0373 (6)	0.0322 (6)	0.0306 (7)	−0.0002 (5)	0.0112 (5)	0.0006 (5)
C11	0.0441 (7)	0.0348 (7)	0.0385 (8)	−0.0018 (5)	0.0133 (6)	−0.0009 (6)
C1	0.0378 (6)	0.0330 (6)	0.0428 (8)	0.0019 (5)	0.0134 (6)	0.0040 (5)
C8	0.0435 (7)	0.0368 (7)	0.0324 (8)	−0.0010 (5)	0.0103 (6)	0.0035 (6)
C12	0.0530 (8)	0.0359 (7)	0.0551 (10)	−0.0045 (6)	0.0179 (7)	0.0042 (7)
C2	0.0521 (8)	0.0465 (8)	0.0471 (10)	−0.0060 (7)	0.0118 (7)	0.0093 (7)
C6	0.0551 (9)	0.0385 (7)	0.0506 (10)	−0.0026 (6)	0.0199 (8)	−0.0019 (7)
C13	0.0727 (12)	0.0511 (10)	0.0503 (11)	−0.0066 (9)	0.0198 (9)	0.0124 (8)
C4	0.0581 (9)	0.0373 (8)	0.0833 (15)	−0.0030 (7)	0.0253 (10)	0.0121 (8)
C3	0.0608 (10)	0.0536 (9)	0.0599 (12)	−0.0052 (8)	0.0161 (9)	0.0203 (8)
C5	0.0623 (10)	0.0363 (8)	0.0720 (13)	−0.0058 (7)	0.0246 (9)	−0.0068 (8)
O3W	0.0807 (10)	0.0829 (10)	0.0325 (7)	−0.0270 (8)	0.0112 (6)	0.0031 (6)

### Geometric parameters (Å, º)

**Table:**

S12—C11	1.7254 (16)	C1—C6	1.398 (2)
S12—C10	1.7744 (15)	C8—H8	0.92 (2)
O1—C10	1.2038 (19)	C12—C13	1.486 (3)
N3—N4	1.3619 (16)	C2—C3	1.390 (2)
N3—C10	1.3804 (19)	C2—H2	0.99 (2)
N3—C9	1.4170 (17)	C6—C5	1.388 (2)
O2—C12	1.210 (2)	C6—H6	0.95 (2)
N4—C11	1.2887 (18)	C13—H13B	0.86 (4)
N1—C7	1.3466 (19)	C13—H13C	0.89 (5)
N1—N2	1.3538 (16)	C13—H13A	0.96 (3)
N1—H1	0.93 (2)	C4—C5	1.372 (3)
N2—C9	1.3229 (19)	C4—C3	1.375 (3)
C7—C8	1.385 (2)	C4—H4	0.92 (2)
C7—C1	1.4724 (18)	C3—H3	0.91 (3)
C9—C8	1.3960 (19)	C5—H5	0.92 (3)
C11—C12	1.489 (2)	O3W—H3A	0.86 (3)
C1—C2	1.387 (2)	O3W—H3B	0.83 (4)
			
C11—S12—C10	88.73 (7)	C9—C8—H8	129.2 (12)
N4—N3—C10	117.57 (11)	O2—C12—C13	124.48 (16)
N4—N3—C9	117.81 (11)	O2—C12—C11	117.62 (16)
C10—N3—C9	124.49 (12)	C13—C12—C11	117.90 (14)
C11—N4—N3	110.27 (12)	C1—C2—C3	120.31 (17)
C7—N1—N2	112.70 (12)	C1—C2—H2	122.2 (13)
C7—N1—H1	130.7 (13)	C3—C2—H2	117.5 (13)
N2—N1—H1	115.8 (13)	C5—C6—C1	120.13 (17)
C9—N2—N1	103.70 (11)	C5—C6—H6	118.6 (12)
O1—C10—N3	126.60 (14)	C1—C6—H6	121.3 (12)
O1—C10—S12	126.50 (12)	C12—C13—H13B	113 (2)
N3—C10—S12	106.89 (10)	C12—C13—H13C	110 (3)
N1—C7—C8	106.73 (12)	H13B—C13—H13C	115 (4)
N1—C7—C1	122.84 (13)	C12—C13—H13A	116.2 (19)
C8—C7—C1	130.42 (14)	H13B—C13—H13A	101 (3)
N2—C9—C8	113.13 (12)	H13C—C13—H13A	101 (3)
N2—C9—N3	117.96 (12)	C5—C4—C3	120.03 (16)
C8—C9—N3	128.89 (13)	C5—C4—H4	120.7 (15)
N4—C11—C12	121.91 (14)	C3—C4—H4	119.3 (15)
N4—C11—S12	116.53 (11)	C4—C3—C2	120.32 (19)
C12—C11—S12	121.52 (11)	C4—C3—H3	122.7 (15)
C2—C1—C6	118.83 (14)	C2—C3—H3	116.9 (15)
C2—C1—C7	119.80 (14)	C4—C5—C6	120.37 (18)
C6—C1—C7	121.37 (14)	C4—C5—H5	124.8 (15)
C7—C8—C9	103.72 (13)	C6—C5—H5	114.8 (16)
C7—C8—H8	127.1 (12)	H3A—O3W—H3B	105 (3)
			
C10—N3—N4—C11	0.25 (18)	N1—C7—C1—C2	−170.77 (14)
C9—N3—N4—C11	176.34 (12)	C8—C7—C1—C2	10.4 (2)
C7—N1—N2—C9	1.40 (16)	N1—C7—C1—C6	9.5 (2)
N4—N3—C10—O1	179.14 (16)	C8—C7—C1—C6	−169.31 (15)
C9—N3—C10—O1	3.3 (3)	N1—C7—C8—C9	0.87 (16)
N4—N3—C10—S12	−0.66 (16)	C1—C7—C8—C9	179.81 (14)
C9—N3—C10—S12	−176.46 (10)	N2—C9—C8—C7	−0.03 (17)
C11—S12—C10—O1	−179.15 (18)	N3—C9—C8—C7	178.50 (13)
C11—S12—C10—N3	0.65 (11)	N4—C11—C12—O2	173.25 (16)
N2—N1—C7—C8	−1.46 (17)	S12—C11—C12—O2	−4.4 (2)
N2—N1—C7—C1	179.50 (12)	N4—C11—C12—C13	−6.8 (2)
N1—N2—C9—C8	−0.80 (16)	S12—C11—C12—C13	175.50 (14)
N1—N2—C9—N3	−179.51 (12)	C6—C1—C2—C3	1.3 (3)
N4—N3—C9—N2	−1.44 (18)	C7—C1—C2—C3	−178.47 (16)
C10—N3—C9—N2	174.35 (14)	C2—C1—C6—C5	−0.6 (2)
N4—N3—C9—C8	−179.92 (13)	C7—C1—C6—C5	179.19 (14)
C10—N3—C9—C8	−4.1 (2)	C5—C4—C3—C2	−0.4 (3)
N3—N4—C11—C12	−177.47 (13)	C1—C2—C3—C4	−0.8 (3)
N3—N4—C11—S12	0.32 (16)	C3—C4—C5—C6	1.1 (3)
C10—S12—C11—N4	−0.59 (13)	C1—C6—C5—C4	−0.7 (3)
C10—S12—C11—C12	177.22 (14)		

### Hydrogen-bond geometry (Å, º)

**Table:**

*D*—H···*A*	*D*—H	H···*A*	*D*···*A*	*D*—H···*A*
N1—H1···O3*W*^i^	0.93 (2)	1.83 (2)	2.749 (2)	173 (2)
O3*W*—H3*A*···N2^ii^	0.86 (3)	1.99 (3)	2.8402 (19)	169 (2)
O3*W*—H3*B*···O1^iii^	0.83 (5)	2.19 (5)	2.995 (2)	163 (4)
C8—H8···O1^iv^	0.93 (2)	2.50 (2)	3.4100 (19)	167.8 (15)

Symmetry codes: (i) *x*−1, *y*, *z*; (ii) −*x*+1, −*y*+1, −*z*; (iii) −*x*+1, −*y*+1, −*z*+1; (iv) −*x*+1, −*y*+1, −*z*+2.
